# Hospitals and Mental Cases

**Published:** 1919-11-29

**Authors:** 


### Hospitals and Mental Cases.

### PSYCHIATRY CLINIC AT CARDIFF.

AT Cardiff Mental Hospital, of which Colonel E. Goodall, R.A.M.C., is general superintendent, a psychiatry clinic is about to be opened in collaboration with the Board of Control. The clinic will treat mental and nervous diseases in their earliest phases, and receive patients without judicial proceedings or certificates for at least six months. The "stigma" of the asylum being removed, patients are expected to come at an early stage of the malady. The provision will be for all classes, but a moderate charge will be made for private cases. An out-patients' department will be associated with the clinic, the actual work being done at the out-patients' department of King Edward VII.'s Hospital. The clinic will also assist the training of students and nurses and aid research.

The building, as planned, will comprise wards for twenty-five patients of each sex, with the necessary staff accommodation. It will also include a lecture-room, library, photo- and radiographic room, psychological laboratory, and a small vegetable garden; and it is proposed to add a few workshops and a gymnasium. The plan of the building will admit of extension later, to accommodate twenty more patients. Provision for fifty patients will require five acres of land. Additions will have to be made to the asylum before long, but by treating mental cases at an early stage at the psychiatry clinic the need for extensions, it is hoped, will be postponed. The institution will be similar to those contemplated at Birmingham and other centres.

